# Platelet adhesion onto immobilized fibrinogen under arterial and venous in-vitro flow conditions does not significantly differ between men and women

**DOI:** 10.1186/1477-9560-5-5

**Published:** 2007-04-26

**Authors:** Robert Loncar, Reiner B Zotz, Christoph Sucker, Aleksandar Vodovnik, Mario Mihalj, Rüdiger E Scharf

**Affiliations:** 1 Institut für Hämostaseologie und Transfusionsmedizin, Heinrich-Heine-Universität, Moorenstr. 5, D-40225 Düsseldorf, Germany; 2 Department of Histopathology, The Calderdale Royal Hospital, HX3 0PW Halifax, UK; 3 Department of Neurology, University Hospital Firule, Split, Croatia

## Abstract

**Background:**

Gender-related differences in incidence of arterial thrombosis have been a focus of interest for years. The platelet integrin αIIbβ3 is primarily responsible for the interaction between platelets and fibrinogen and consecutive thrombus growth. In this study, we evaluated platelet adhesion onto immobilized fibrinogen under venous and arterial flow conditions in men and women.

**Methods:**

Platelets in whole anticoagulated blood were labelled with the fluorescence dye Mepacrine and perfused through the rectangular flow chamber over glass cover slips coated with fibrinogen (shear rates of 50 s^-1^, 500 s^-1 ^and 1500 s^-1^). A fluorescence laser-scan microscope was used for visualisation and quantification of platelet adhesion at 15 seconds, 1 and 5 minutes after the start of perfusion.

**Results:**

During perfusion, the platelet adhesion linearly increased in regard to exposition time and shear rate. After five minutes of perfusion the platelet adhesion onto immobilized fibrinogen showed no significant gender related difference, neither at 50 s^-1 ^nor at 500 s^-1 ^and 1500 s^-1 ^(p > 0.05), respectively. No significant difference in platelet adhesion onto immobilized fibrinogen, in regard to the menopausal status, was either observed (p > 0.05).

**Conclusion:**

In our in vitro experimental system, hormonal differences between men and women did not influence platelet adhesion onto immobilized fibrinogen, neither under venous nor under arterial rheological conditions.

## Background

Ischemic heart and cerebrovascular disease are leading causes of morbidity and mortality in the western world and are steadily increasing incidence in the third world as well [1,2]. Epidemiological studies [3] indicate that these diseases result from complex interactions between genetic susceptibility factors, chronic environmental influences (e.g. hormonal imbalance, smoking, obesity) and established, intercurrent disorders (e.g. diabetes, hypertension, dyslipidemia, or hyperhomocysteinemia). The most devastating complication of these disorders is acute myocardial infarction or stroke resulting from the formation of occlusive thrombus at the site of ruptured atherosclerotic plaque [1-4]. Platelet-dependent thromboembolism is an underlying mechanism of arterial thrombosis and the critical role of platelets in this process is now widely accepted [1,5]. Its participation in the arterial thrombosis is centered on their adhesive properties and their ability to respond to stimuli with rapid activation and, as end effect, aggregation [5,6].

It has been well known that the incidence of arterial thrombotic events in women is generally lower compared to that in men. It was our initial point to speculate about beneficial effects of female sexual hormons in regard to the incidence of arterial thrombosis and one of the reasons for non-critical and non-selective inducing estrogen replacement therapy (ERT). Although several studies have demonstrated that postmenopausal women who take ERT show decreased incidence of arterial thrombosis compared to those without ERT [7], there has been no clear epidemiological evidence for decrease in a thrombotic risk. All these reports of beneficial effects of estrogen are based on the retrospective studies and a small number of controlled clinical trials. Recently conducted clinical trials have shown ERT could also be associated with adverse arterial vascular events [8-10]. It seems that risk and benefit effect of estrogen is also dose dependent. However, the possible mechanism and efficiency of estrogen mediated antithrombotic or prothrombotic action is still a controversial issue.

Arterial thrombosis is initiated by platelet activation, but the in-vivo effects of estrogens on a platelet function have not been well understood. Platelet membrane integrin αIIbβ3 (glycoprotein GP IIb–IIIa) has an important role in platelet adhesion and aggregation through binding of a variety of circulating and immobilized ligands including fibrinogen under different shear stress conditions [5,6,11,12]. However, when platelets are activated by various stimuli, the platelet αIIbβ3 undergoes a conformational change and provides a high-affinity binding site for a soluble fibrinogen. The fibrinogen acts as a bridging molecule between pairs of αIIbβ3 molecules in adjacent activated platelets [5,6,13]. On the other side, the interaction between immobilized fibrinogen and platelets does not require previous platelet activation or conformational changes of platelet αIIbβ3 and has been shown to occur even in the presence of platelet inhibitors [5]. An immobilized fibrinogen can be found on the injured surface of vascular endothelium, atherosclerotic plaques and vascular prostheses, where it is the major ligand mediating platelet thrombus growth on artificial surfaces [5,6]. This study, conducted under strictly controlled experimental venous and arterial environmental conditions, was undertaken to determine whether platelet adhesion in healthy individuals, mediated through the integrin αIIbβ3, was depending on the gender-related hormonal status.

## Methods

The study was conducted on 28 age-matched and healthy blood donors (14 women and 14 men). The mean age was 44 ± 12 years. In the preceding 14 days none of donors had taken any medication. None of enrolled women was on oral contraceptives or ERT and had no proven anamnestic evidence of hormonal disbalance. In each participant screening tests of coagulation, factors of coagulation and different biochemical parameters (Table 1) were assessed.

**Table 1:** Descriptive statistical data related to participant's gender, age, haemostaseology and biochemical parameters

Parameter	Female	Male	p
Age	46 ± 12	41 ± 10	ns
Htc	37 ± 3	41 ± 2	0.014
Fibrinogen	285 ± 71	231 ± 60	ns
FII	146 ± 20	124 ± 20	0.037
FV	141 ± 22	125 ± 27	ns
FVII	140 ± 46	116 ± 32	ns
FVIII	174 ± 109	119 ± 34	ns
VWF-Activity	179 ± 105	172 ± 103	ns
VWF-Ag	164 ± 69	133 ± 74	ns
FIX	126 ± 24	116 ± 27	ns
FX	138 ± 34	122 ± 24	ns
FXI	111 ± 25	101 ± 32	ns
FXII	113 ± 16	98 ± 8	0.029
FXIII	127 ± 16	108 ± 19	0.04
Protein C	120 ± 21	112 ± 16	ns
Protein S	92 ± 18	105 ± 15	ns
Plasminogen	110 ± 14	108 ± 12	ns
Trigliceride	156 ± 99	197 ± 110	ns
Cholesterol	199 ± 36	190 ± 37	ns
LDL	124 ± 28	108 ± 25	ns
HDL	52 ± 18	39 ± 10	ns
CRP	0.6 ± 0.5	0.5 ± 0.6	ns
Fe	60 ± 34	101 ± 49	ns
Ferritine	10 ± 7	26 ± 19	ns
Homocysteine	9 ± 4	10.5 ± 4	ns
ATIII	104 ± 8	99 ± 7	ns
Thr	304 ± 50	243 ± 51	0.019

p = level of statistical significance (two-side)

ns = no significance

Excessive smokers, overweight donors, donors with a family history of neoplastic, arterial or venous vessels disease were excluded from the study. Experimental protocols were reviewed and approved by the local ethics committee and meet the standards of the Declaration of Helsinki.

### Blood preparation

Blood taken from the cubital vein was immediately anticoagulated with PPACK (D-Phenylalanyl-L-prolyl-L-arginine chloromethyl ketone) at a final concentration of 40 μM. Platelets were labelled with the fluorescence dye Mepacrine (quinacrine dihydrochloride, final concentration 10 μM; Sigma Chemical, 60 min at 37°C). The dye immediately accumulates in the delta granules of platelets without influencing a platelet physiology [6]. Blood was used within two hours of its withdrawal.

Binding specificity of platelets to immobilized fibrinogen was tested with two additional experimental designs. In the first experimental set, a specificity of platelet adhesion onto immobilized fibrinogen was tested in flow experiments using glass coverslips co-coated with bovine serum albumin (BSA, 5 μg/mm^2^, n = 3). In the second set of experiments (n = 3) a blood was additionally incubated with Abciximab (c7E3, 4 μg/mL for 10 min, 37°C) and perfused over fibrinogen coated coverslips. c7E3 Fab is a chimeric human/mouse Fab fragment derived from the murine monoclonal 7E3 antibody (c7E3, Centocor Inc. Leiden, Niederland) that binds selectively to the αIIbβ3 [14].

Screening parameters and factors of coagulation were assessed with commercial hemostatic high-speed analyzers and kits for clotting as well as chromogenic and immunologic coagulation assays (BCT^® ^and BCS^®^, Dade-Behring, Marburg Germany). Both systems measure coagulation capabilities of plasma from blood collected using a 3.8% sodium citrate as anti-coagulant.

### Genotyping

All subjects were genotyped for the β3 polymorphism of αIIbβ3 (Leucine-Proline substitution at amino acid 33) by polymerase chain reaction amplification of genomic DNA followed by restriction enzyme digestion (polymerase chain reaction-restriction fragment length polymorphism), as published previously [15].

### Preparation of fibrinogen-coated cover slips

Suspensions of human fibrinogen (Sigma-Aldrich) were prepared as previously described [6]. Glass cover slips (24 × 50 mm) were coated with 50 μl of fibrinogen solution (2,5 mg/mL). The cover slip was placed in a humid environment (60 min at 37°C) to allow the protein to adhere to the glass surface. The coated cover slips were rinsed with 10 mL of 50 mmol/L phosphate buffered saline (pH 7.35) to remove non-adherent fibrinogen and were placed into the flow chamber. Fibrinogen density on the glass surfaces was calculated to be 0.1 μg/mm^2^. For control experiments, three cover slips were coated with bovine serum albumin. Glass surfaces density of bovine serum albumin was calculated to be of 5 μg/mm^2^.

### Flow chamber and laser-scan microscopy

Platelet adhesion rate onto fibrinogen-coated glass cover slips was conducted in the rectangular flow chamber [16] under linear shear rate of 50 s^-1^, 500 s^-1 ^and 1500 s^-1^, respectively.

One side of the parallel flow chamber was formed by a fibrinogen coated glass cover slip with a flow path height of 50 μm, determined by Teflon gasket. Assembled flow chambers were filled with phosphate buffered saline (pH 7.35). According to the Newtonian fluid axiom, a shear stress is constant and dependent on the flow rate. Shear rate of 50 s^-1 ^represents venous environment, share rate of 500 s^-1 ^mimics wall shear rate of larger arteries; shear rate of 1500 s^-1 ^represents a typical arteriolar shear rate as well as shear rate in moderate arterial stenosis [17]. An epifluorescence laser-scan microscope (Axiovert 100 M, Carl-Zeiss, Jena, Germany) allowed a real-time visualisation of labelled platelets during perfusion through the chamber. To assess the time-course of platelet adhesion, series of images (five images per series, 0,7 s per image) were recorded at 0, 1 and 5 minutes. The zero time point represents 15 seconds after the start of perfusion (Perfusor, B. Braun, Meslingen, Germany). Image analysis was performed using the ImageJ software (version 1.26t, NIH, USA). This program allows evaluation of platelet-surface interaction, consecutive aggregation and evaluation of thrombus generation at one exactly defined area in each image. A single frame image corresponds to the area of 980 × 980 μm. The stable attached platelets (expressed as fluorescence) were defined as those, which remain at their initial adhering position in the first and second image (time frame of 0.7 sec). Platelets were considered to move on the surface when exhibiting spatial displacement greater than a diameter of one platelet. To estimate the motion, a series of 5 images (time frame 0.7 sec) at one time point was made. Using ImageJ software, images were digitalized and a threshold was applied to distinguish platelet from background. The first two consecutive frames in a series were superimposed using the logical AND function. The resulting image represented only the overlapping areas of single platelets at two different time points.

### Calculations and statistics

Data in this study are given as mean values ± SD. Absolute fluorescence was expressed as arbitrary units (pixel units) and represents sum of fluorescence of each thrombus or individual adherent platelet in one defined area. Only platelets showing a stable adherence during one series of image were taken into calculation. Platelet adhesion was calculated using the logic function of the applied software (ImageJ) and represents stable adhesion of platelets between the first and second image. To reduce the influence of inter-individual variation, data were normalized (absolute fluorescence recorded after five minute of perfusion was divided by the recorded fluorescence after one minute) and expressed as a relative adhesion. The relative adhesion represents an increase of absolute fluorescence as a function of time.

Differences between experimental groups were tested by Student's t-test (two-sided). Regression analyses were based on individual measurements using Spearman's rank correlation coefficient. Statistical analyses were performed using SPSS for Windows, version 6.0.1. *P*-value of less than 0.05 (two-sided) was taken to indicate a significant difference.

## Results

A specificity of binding of platelets to immobilized fibrinogen in experimental in vitro system was tested with blood preincubated with Abciximab. A perfusion of five minutes (1500 s^-1^) of Abciximab preincubated blood over fibrinogen-coated cover slips showed no significant adherence of platelets (absolute fluorescence of stable adherent platelets: 168 U ± 35 U vs. 50535 U ± 21552 U in control experiments, p < 0.05). Similarly, perfusion over BSA-coated glass cover slips falled to show significant platelet adherence.

The baseline characteristics of the study participants are summarized in the Table 1 Age average was 44 ± 12 years and did not significantly differ between men and women (41 ± 10 vs 46 ± 12 years, p > 0.05), respectively. Biochemical parameters and factors of coagulation were within the normal range in all participants. Men showed a significantly higher value of haematocrit and women higher platelet count. A significant difference between men and women was observed for Fll, FXII and FXIII. Additional analysis indicated that these differences did not influenced platelet adhesion onto immobilized ligand. All other parameters of plasmatic haemostasis as well as biochemical parameters did not differ significantly.

During the perfusion, a continous increase in platelet adhesion on fibrinogen-coated surfaces, in function of time and in shear rates, was observed. At shear rate of 50 s^-1^, platelet adhesion from 15 sec to 5 minutes increased 3.8-fold (from 2686 ± 1606 U to 10344 ± 4846 U), at 500 s-1 11-fold (from 3417 ± 1623 U to 38227 ± 16032 U) and at 1500 s^-1 ^platelet adhesion increased 17-fold (from 2948 U ± 1585 U to 50535 U ± 21552 U), respectively. Parameters of plasmatic haemostasis as well as other biochemical parameters, which were in the normal range, did not correlate with the platelet adhesion. Spearman's correlation coefficients between platelet adhesion and examined variables were not significant (p > 0.05). After 5 minutes of perfusion, an analysis of the platelet adhesion onto the immobilized fibrinogen, expressed as absolute fluorescence, showed no significant difference related to the gender, neither at 50 s^-1 ^nor at 1500 s^-1^, p > 0.05. Descriptive statistical data of platelets adhesion in regards to the gender and shear rate are presented in the Table 2.

**Table 2:** Platelet adhesion (mean ± SD) onto immobilized fibrinogen expressed as absolute fluorescence (AU) related to the gender, shear rate and perfusion time.

**Shear rate and perfusion time**	**Platelet adhesion, AU**	
venose shear rate, 50 s^-1^	males	Females

15 sec	2408 ± 1697	2965 ± 1537
1 min	5857 ± 2581	7235 ± 3387
5 min	9364 ± 4588	11324 ± 5114
arterial shear rate 500 s^-1^		
15 sec	3426 ± 1744	3408 ± 1578
1 min	9949 ± 3580	12915 ± 8963
5 min	31980 ± 11246	44474 ± 18095
arterial shear rate 1500 s^-1^		
15 sec	2503 ± 1227	3392 ± 1826
1 min	12015 ± 4571	14161 ± 5018
5 min	47665 ± 18504	53404 ± 24797

Taking into consideration that platelet adhesion and platelet detachment after initial adhesion is a dynamic process, dependent from shear rate and perfusion time and to avoid inter-individual variation data were normalized (see Statistics).

The results of statistical evaluation of normalized data at typical venous (50 s^-1^) and arterial (1500 s^-1^) rheological conditions was shown in the Table 3. A growth of stable adherent platelets was expressed as relative adhesion and evaluated in two phases, initial and late. The initial phase represents growth of stable adherent platelets within first 45 sec of perfusion. The late phase represents growth of stable adherent platelets between first and fifth minute of perfusion. Platelet relative adhesion showed slightly higher trend at both shear conditions and in both time frames, in men compared to women (in contrast to the trend observed when expressed as an absolute fluorescence). This difference showed no statistical significance (p > 0.05).

**Table 3:** Relative platelet adhesion onto immobilized fibrinogen related to the gender and shear rate.

Gender	R. a. 50 s^-1^		R. a. 1500 s^-1^	
	initial	late	initial	Late

Male	4.72 ± 2.05	1.94 ± 1.31	5.39 ± 2.40	4.09 ± 1.40
Female	3.02 ± 2.15	1.60 ± 0.39	4.56 ± 1.44	3.85 ± 1.31

R.a. (Relative adhesion) = absolute fluorescence recorded after one minute of perfusion was divided by fluorescence recorded 15 sec after start of perfusion (initial). Absolute fluorescence recorded after five minute of perfusion and divided with fluorescence recorded one minute after start of perfusion was marked as late.

However, in our study, platelet adhesion did not exhibit a significant difference between men and women (p > 0.05.), neither expressed as an absolute adhesion nor expressed as a relative adhesion. Further evaluation showed no significant relationship between examined variables (see Table 1), neither in men nor in women. None of indicated parameters significantly correlated with platelet adhesion.

Nevertheless, no significant correlation was found between the platelet adhesion and age, when related to gender (p > 0.05).

**Figure 1:**
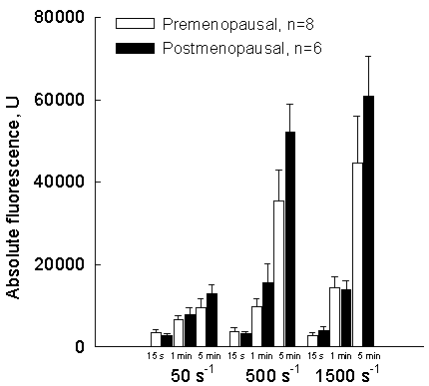
Platelet adhesion onto immobilized fibrinogen, expressed as absolute fluorescence in pre- and postmenopausal women (n represents the number of tested subjects). Platelet adhesion at typical venous (50 s^-1^) and arterial (500 s^-1 ^– 1500 s^-1^) shear rates was tested at three different time points (15 sec, 1 min and 5 min). At each time point of perfusion a stack of 5 images was collected and analyzed. Finally 42 perfusion experiments were conducted and 630 images were analyzed.

Taking into consideration expected significant differences in hormonal status between pre- and postmenopausal women, further statistical evaluation was focused on these two subgroups. A relationship between platelet adhesion in all examined shear rates in pre- and postmenopausal women are summarized in the Figure 1, showing platelet adhesion expressed as the absolute fluorescence. In the Figure 1, a platelet adhesion at typical venous (50 s^-1^) and arterial (500 s^-1 ^– 1500 s^-1^) shear rates at three different time points (15 sec, 1 min and 5 min), in pre- and postmenopausal women, was presented. At all shear rates and at each time point (excluded 15 sec at 50 s^-1^), one slightly higher platelet adhesion was observed in postmenopausal women, without statistical significance. To further characterize these subgroups, all parameters presented in the Table 1 were evaluated. No significant difference between examined parameters was observed in regard to the menopausal status. Sperman's correlation coefficient between platelet adhesion and each parameter was tested and did not differ significantly, p > 0.05.

Experiments with immobilized collagen were conducted together with experiments with fibrinogen (data are not showed) and the relationship between gender related platelet adhesion onto immobilized collagen in regard to the shear rate and time of perfusion was carefully evaluated. Between males and females, neither significant difference nor constant trend could be found in regard to the platelet adhesion. The analysis of normalized data obtain similar results.

## Discussion

After the menopause, the risk of cardiovascular disease in women increases to equal the risk in men, which makes a rationale for implementation of estrogen replacement therapy (ERT). Paradoxically, the use of estrogen itself could also be associated with a thromboembolic disease in women on oral contraception as well as in men treated with diethylstilbestrol for prostate cancer [18,19]. Although the estrogen has been clinically available for more than six decades, there has been some confusion defining the risks and benefits of menopausal ERT [8-10]. Reagrding the central role of platelets in arterial thrombosis, it was hypothesized that a hormonal status could have a significant impact on platelet adhesion. Following the observation of gender-dependent difference in incidence of arterial thrombosis, it was of interest to evaluate whether platelet adhesion under dynamic rheological condition is gender-related or not. However, in our study, platelet adhesion did not exhibit a significant difference between men and women (p > 0.05.), neither expressed as an absolute adhesion nor expressed as a relative adhesion. As shown in the results, in experiments with immobilized fibrinogen, women surprisingly showed consistently higher adherence at each shear rates and at each time point but without statistical significance (Table 2). This difference was constant under venous flow conditions (50 s^-1^, average 22%) and continuously declined along the perfusion time under arterial flow conditions (at 15 sec 25% and at 5 min 11%). Postmenopausal women showed a slightly higher platelet adhesion compared to premenopausal but without statistical significance. No significant correlation was found either between platelet adhesion and age, related to gender either.

In two very interesting studies, Miller et al. explored the adhesive properties of platelet under static and dynamic flow condition in regard to the gender and menopausal status [20,21]. In static experiments the authors found out that platelet from women showed a higher adhesion activity than those in men. This difference increased in postmenopausal women. Simultaneously, women on ERT showed a significantly increased platelet adhesion rate compared to the premenopausal or postmenopausal women without ERT. In contrast to static experiments, in the flow studies conducted at 25 s^-1 ^(extreme low flow) with platelets ex-vivo preincubated with estrogen, a decreased rate of platelet adhesion onto fibronectin was observed [20].

Boudoulas et al [22] tried to explain this controversy of ERT indicating that the benefit of estrogen could be impacted in regard to the HPA-1 polymorphism. In the aggregation study, the author described that only platelets with HPA-1b allele had shown the benefits of estrogen supplementation (reduced aggregation).

These findings could not be confirmed in our study. Firstly, we did not found any significant difference in platelet aggregation in regard to the HPA-1 polymorphism and secondly, no significant difference in platelet adhesion was observed between women and men in regard to the HPA-1 polymorphism neither at venous nor at arterial flow conditions.

Antoher possible explanation was recently given by Tong et al [23]. The author reported that reduction activity of estrogen sulfotransferase (EST), due to increasing estrogen concentration and its bioavilability induce prothrombotic state. In animal model with generated EST knockout mice (Sult 1-/-) a significantly higher incidence of placental thrombosis and spontaneous fetal loss, caused by an estrogen excess, was observed. The additional analysis of knockout mice showed induced focal (placental) tissue factor expression as well as increased level of procoagulant factors VII, X, XII and XIII. This phenomenom was abolished through administration of low molecular weight heparin and/or related antiestrogen substance. It seems that this prothrombotic effect is restricted to the venose thrombosis, while platelets reactivity was only moderate enhanced.

## Conslusion

Platelet adhesion onto immobilized fibrinogen under arterial and venous in-vitro flow conditions did not exhibit a significant difference between men and women. Platelet adhesion between women and men did not differ significantly with regard to the HPA-1 polymorphism.

## Competing interests

The author(s) declare that they have no competing interests.

## Authors' contributions

RL: initiated the study, designed, recruited participants, coordinated and drafted the manuscript.

RBZ: performed the statistical analysis.

CS: recruited participants and helped to draft the manuscript.

AV: participated in the design of the study and helped to draft the manuscript.

MM: participated in analysis of digital imaging and statistical evaluation.

RES: initiated and coordinated the study.

All authors read and approved the final manuscript.
